# *PTGDR* expression is upregulated through retinoic acid receptors (RAR) mechanism in allergy

**DOI:** 10.1371/journal.pone.0215086

**Published:** 2019-04-15

**Authors:** Asunción García-Sánchez, Elena Marcos-Vadillo, Catalina Sanz, Miguel Estravís, María Isidoro-García, Ignacio Dávila

**Affiliations:** 1 Department of Biomedical and Diagnostic Sciences, University of Salamanca, Salamanca, Spain; 2 Institute for Biomedical Research of Salamanca, IBSAL, Salamanca, Spain; 3 Asthma, Allergic and Adverse Reactions (ARADyAL) Network for Cooperative Research in Health of Instituto de Salud Carlos III, Salamanca University Hospital, Salamanca, Spain; 4 Department of Clinical Biochemistry, Salamanca University Hospital, Salamanca, Spain; 5 Department of Microbiology and Genetics, University of Salamanca, Salamanca, Spain; 6 Department of Medicine, University of Salamanca, Salamanca, Spain; 7 Department of Allergy, Salamanca University Hospital, Salamanca, Spain; Seoul National University College of Pharmacy, REPUBLIC OF KOREA

## Abstract

Functional studies suggest that promoter polymorphisms of the Prostaglandin D Receptor (*PTGDR*) gene can be involved in asthma. All-trans Retinoic acid (ATRA) has also been linked to allergic diseases. We have previously described the *PTGDR* promoter activation mediated by ATRA through response elements (RARE) at position -549T> C. In this study we aimed to analyze the effect of retinoic acid (RA) on the expression of *PTGDR*, the production of cytokines as well as to evaluate the binding of RA receptors to RA-Response Elements (RARE) sequences. A549 cells were transfected with vectors carrying different *PTGDR* haplotypes and treated with all-Trans Retinoic Acid (ATRA). *PTGDR* expression was measured by qPCR. Chromatin Immunoprecipitation assays (ChIP) were performed in ATRA stimulated KU812 cells and in PBMCs of patients carrying CTCT, CCCC or CCCT haplotypes. In addition, a broad panel of cytokines was analyzed by cytometric bead assay in A549 cells. The expression of *PTGDR* increased in A549 cells transfected with *PTGDR*-variants. The CCCC haplotype showed a significantly higher expression compared with CTCT. However, we found that RA up-regulated *PTGDR* expression through RARα mainly in the CTCT variant. Experiments on PBMCs from allergic patients carrying the -549T and -549C variant of the *PTGDR* promoter after ATRA and RAR antagonist administration confirmed the modulation of *PTGDR* by ATRA. The cytokine analysis showed that IL4 and IL6 levels were significantly increased in A549 cells transfected with *PTGDR*. In addition, ATRA treatment decreased the levels of IL4, IL6 and TNFα in A549 cells, whereas it increased IL4 and TNFα levels in *PTGDR*-transfected cells. We observed genetic differences in the regulation of *PTGDR* by ATRA that could contribute to the phenotypic differences observed in allergic patients. Our findings showed that RAR modulation by *PTGDR* might have an impact on Th2 responses, suggesting that RAR could be a potential therapeutic target in allergic inflammation.

## Introduction

Asthma is a chronic inflammatory disease affecting more than 358 million people [1]. The inflammatory response in asthma involves the participation of the respiratory epithelium, the innate immune system and the adaptive immunity [2,3]. Prostaglandin D2 (PGD2) is the major cyclooxygenase (COX) metabolite of arachidonic acid produced in response to allergens and has been proposed as a mast cell activation marker [4]. There are two types of transmembrane receptors of PGD2, the D prostanoid receptor, known as PTGDR, DP or DP1, and the chemoattractant receptor-homologous expressed on Th2 (CRTH2) or DP2 [4,5]. Several *PTGDR* polymorphisms have been previously reported [4,6–8]. Promoter variants have shown a different binding of transcription factors that controls the expression of PTGDR, and this could be related to the development of asthma susceptibility [4,6].

Epidemiological studies have related vitamin A levels [through its active metabolite, the all-trans (AT)-retinoid acid (RA)] with the prevalence of allergic diseases [9]. Some studies have associated vitamin A deficiency with an increase frequency of atopy [9,10], although others have reported that vitamin A supplementation associated with increased airway hyperresponsiveness [11,12]. Dawson et al reported that ATRA promotes the synthesis of the human type 2 cytokines IL4, IL5 and IL13 while decreasing IFNγ and, TNFα expression, and IL12 synthesis in activated human T-cells [13]. RA has previously been associated with the prostaglandin pathway. ATRA has been shown to induce an increase of the expression of cyclooxygenase 2 (COX-2), one of the cyclooxygenases involved on PGD2 synthesis, suggesting ATRA as a main regulator of COX-2 expression [14]. In addition, the synthesis of PGD2 is mediated by the lipocalin-type prostaglandin D synthase (LPGDS), which is a retinoid transporter able to bind ATRA [9]. RA activates nuclear retinoic acid receptors (RARα, β, γ), which dimerize with retinoid X receptors (RXRα, β, γ) and function as ligand-dependent transcriptional regulators by binding to RA response elements (RARE) on target genes [15,16]. We have previously shown that RA activates the *PTGDR* promoter; in addition, we have identified RAREs in the promoter region, and demonstrated that some transcription factor motifs were affected by genetic variants [17]. Considering our previous results, the objective of this study was to deeper explore the regulatory mechanisms involved in the expression of *PTGDR* by ATRA and its effect on cytokine production. In addition, we aimed to evaluate the role of different factors involved in this regulation, like promoter polymorphisms and the different nuclear retinoic acid receptors.

## Materials and methods

### Subjects

The study included 6 adult patients (3 males and 3 females) sensitized to pollen that had been evaluated and diagnosed by allergists. All of them signed an informed written consent. The study was approved by the Ethics Committee of Clinical Investigation of the hospital (PI 1/07/2013).

### Isolation of peripheral blood cells

Peripheral Blood Mononuclear Cells (PBMC) were obtained by venous puncture on Lithium Heparin vacuum tubes. Blood was centrifuged on Ficoll-Paque (GE-Healthcare Life Science, Chicago, IL, USA) density gradients. Interphase cells were washed three times with Dulbecco’s phosphate-buffered saline (PBS, GIBCO-Thermo Fisher Scientific, Whaltham, MA, USA).

### Cell culture

Isolated PBMCs were resuspended at a concentration of 10x10^6^ cells/ml in complete medium, consisting of RPMI 1640 supplemented with 2mM L-glutamine, 1% penicillin-streptomycin (P/S) (GIBCO-Thermo Fisher Scientific, Waltham, MA, USA) and 10% autologous human serum from each patient and cultured for 48h in an incubator at 37°C and 5% CO2.

A549 and KU812 cells (Sigma-Aldrich, Saint Louis, MO, USA) were maintained in RPMI-1640, 2mM L-Glutamine, 10% heat-inactivated Fetal Bovine Serum (FBS), 1% penicillin-streptomycin (P/S) (GIBCO-Thermo Fisher Scientific, Waltham, MA, USA) in an incubator at 37°C and 5% CO_2_. ATRA (Sigma-Aldrich, Saint Louis, MO, USA), pan-RAR antagonist (AGN 193109), and RARβ antagonist (CD 2665) (Santa Cruz Biotechnology, Dallas, TX, USA) stock solutions were made in DMSO and added to cultures at 1μM final concentration when required. Cell cultures were pre-treated with antagonists for 1h before treatment with ATRA.

### Identification of *PTGDR* promoter variants

DNA was isolated using a MagNA Pure Compact device (Roche Applied Science, Mannheim, Germany), PCR amplification were performed using 5’-CTC AGT TTC CTC GCC TAT GC-3’, and 5’-GAG TGA AGG CTG CGG AAG GG-3’. Amplicons were cleaned with Ilustra-Exo-Pro-Star 1-Step (GE-Healthcare Life Science, Chicago, IL, USA) and sequenced in an ABI-Prism Genetic Analyzer (Applied Biosystems, Foster City, CA, USA).

### Expression vector and plasmid construction

The wild-type *PTGDR* cDNA (NM_000953) was cloned in pCMV6-entry (OriGene, Rockville, MD, USA). The constitutive cytomegalovirus promoter of pCMV6-*PTGDR* was replaced by each one of the four promoter sequences of *PTGDR* haplotypes: CTCT, CCCT, TCCT and CCCC (-613C>T, -549T>C, -441C>T and -197T>C positions) [17,18]. Sequences were confirmed by sequencing and transformed into SoloPackGold Competent cells (*E*. *coli*) (Agilent Technologies, Santa Clara, CA, USA). Plasmid DNA was purified with a Maxiprep kit (Qiagen, Hilden, Germany) and verified by sequencing.

### Transient expression transfections

Twenty-four hours before the transfection, A549 cells (50–70% confluent) were plated in RPMI serum free. Transient transfections were carried out with 500ng of pCTCT-*PTGDR*, pCCCC-*PTGDR*, pCCCT-*PTGDR*, pTCCT-*PTGDR*, or pUC18 as carrier DNA (control) and Lipofectamine 2000 reagent (Invitrogen, Carlsbad, CA, USA). After 5h, the medium was replaced by RPMI, 1% FBS. The transfected cells were divided into: basal cells (without treatment), ATRA cells (1μM ATRA) and DMSO cells and incubated for 24 and 48h. The experiments were triplicated, and each sample was performed in triplicate.

### Expression assays

Total RNA isolation, RT-PCR and qPCR were performed as described in detail in the Method Section of the S1 Appendix. Briefly, mRNA was retro-transcribed, and relative qPCR was performed using SYBR-Green-I-Master in a LightCycler480 (Roche Applied Science, Mannheim, Germany). Calculations were made by the comparative Ct method [19]. All procedures were performed following MIQE guidelines [20].

### Chromatin Immunoprecipitation (ChIP) Assay

ChIP was performed using EZ-Magna ChIP A kit (Millipore, Burlington, MA, USA) on KU812 cells and human PBMCs isolated from subjects bearing the *PTGDR* haplotypes [17]. Immunoprecipitated DNA using RARα and RARβ antibodies (Santa Cruz Biotechnology, Dallas, TX, USA) and input samples were subjected to qPCR using primers spanning the -549C/T *PTGDR*-promoter. Additional details are provided in the Method Section of the S1 Appendix.

### Cytokine analysis

Cell supernatants were harvested, centrifuged (400g, 10 min), and stored at -80°C. Cytokine levels were determined using the *Bio-Plex Pro Human Cytokine standard 27-plex*, *Group I* (Bio-Rad, Hercules, CA, USA). Fluorescence was measured by Luminex 100IS (Bio-Rad, Hercules, CA, USA) with Bio-Plex High-throughput fluidics system, powered by the Luminex X-Map Technology (Luminex, Austin, TX, USA). Data were acquired and processed by the Bioplex Manager Software version 4.1.1 (Bio-Rad, Hercules, CA, USA). A preliminary assay showed that 48h was the optimal time for the determination of most cytokines. Each sample was analyzed in triplicate. In addition, expression analysis of cytokine mRNAs after ATRA treatment was performed in A549 cells as previously described.

### Statistical analysis

Data analysis was performed using pairwise comparison by analysis of variance (ANOVA), unpaired one-sample t-test, Kruskal Wallis and Pearson’s correlation coefficient using the SPSS Software (version 23) (IBM, Armonk, NY, USA). Data were representative of at least three independent experiments. A P<0.05 was considered significant.

## Results

### Genetic variants modulate the expression of *PTGDR*

**Fig 1:**
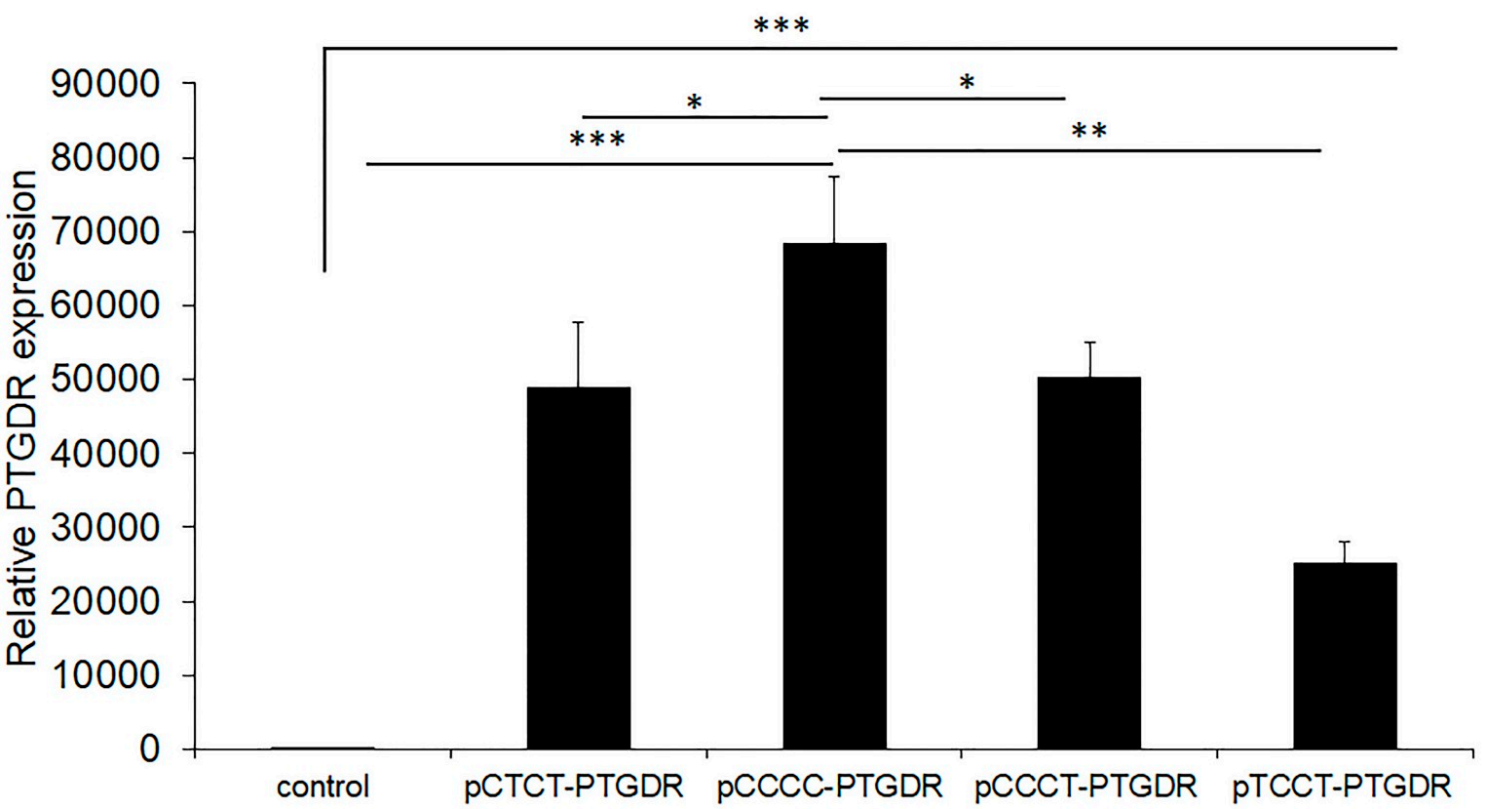
Quantitative real-time PCR analysis of *PTGDR* gene expression. A549 cells were transfected with pCTCT-, pCCCC-, pCCCT- or pTCCT-*PTGDR* expression vectors and with carrier DNA (control condition). Cells were collected after 24h followed by qPCR analysis of *PTGDR* gene. *PTGDR* gene expression was normalized to *GAPDH* mRNA levels. (***p<0.001 for transfected cells with the haplotypic variants versus control. (** P<0.01, for the CCCC-*PTGDR* transfected cells versus TCCT-*PTGDR*, and * P<0.05 versus CTCT and CCCT respectively).

To analyze whether there were changes in the expression of *PTGDR* related to promoter variants, we performed qPCR on A549 line cells transfected with 700 bp fragments of the *PTGDR* promoter carrying CTCT, CCCC, CCCT or TCCT (positions -613C>T, -549T>C, -441C>T and -197T>C, respectively) haplotype variants. Supernatants and cells were collected 24 and 48h after transfection. Transfected cells showed a significant increase in *PTGDR* mRNA expression relative to control cells, detecting most significant differences after 24h (P<0.001) (Fig 1). The *PTGDR* expression after normalization was given as fold increments. Considering control cells = 1, we found: CTCT: 48,990±8,622; CCCC: 68,396±8,943; CCCT: 50,194±4,900; TCCT: 25,206±2,805. The CCCC haplotype showed the highest *PTGDR* expression, whereas the TCCT haplotype showed the lowest (P<0.001 for CCCC vs. TCCT; P<0.05 for CCCC vs. CTCT) (Fig 1).

### The expression of *PTGDR* increased significantly with retinoic acid and is dependent on promoter variants

**Fig 2:**
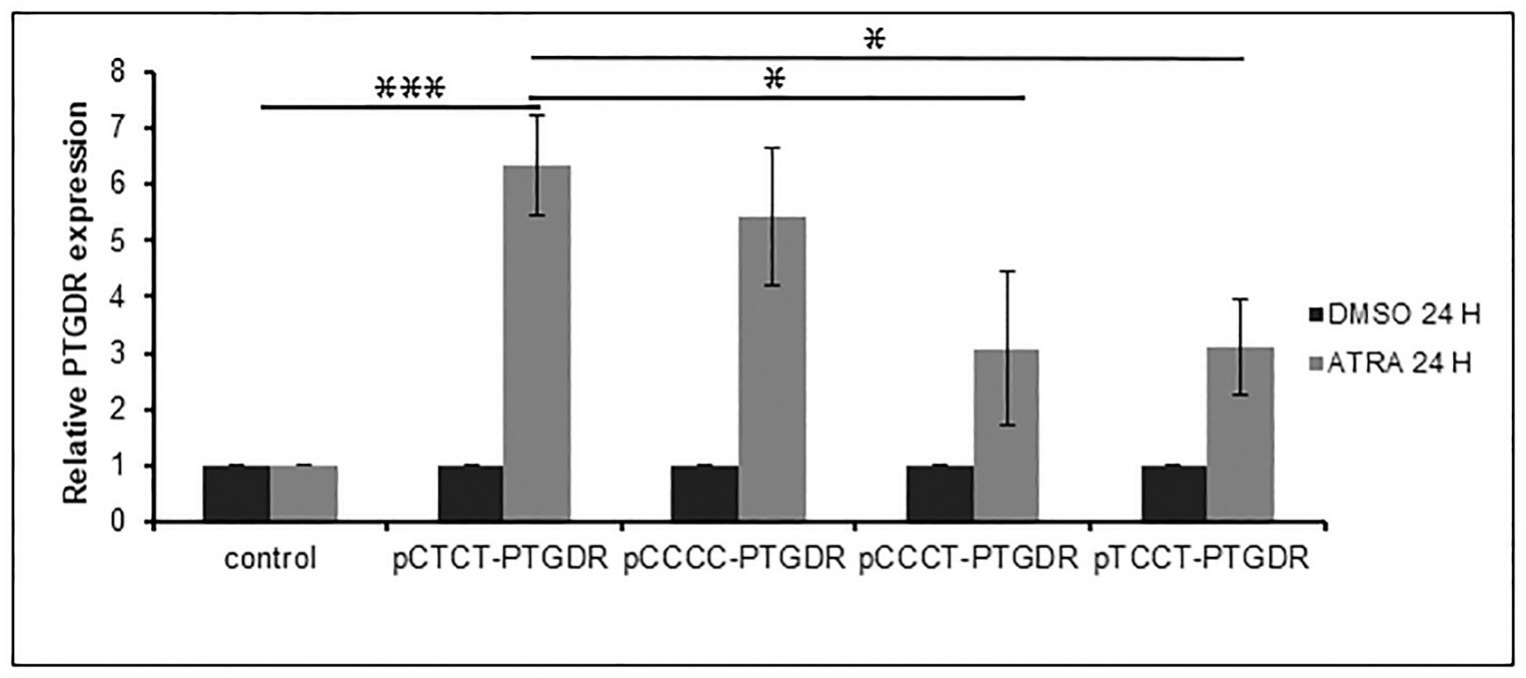
Quantitative real-time PCR analysis of *PTGDR* gene expression after ATRA or DMSO treatment. A549 cells transfected with pCTCT-*PTGDR*, pCCCC-*PTGDR*, pCCCT-*PTGDR* or pTCCT-*PTGDR* expression vectors. and with carrier DNA (control condition) were treated with 1μM ATRA or DMSO and collected after 24 followed by qPCR analysis as above mentioned. *** P<0.001 for transfected cells treated with ATRA versus DMSO at 24 (Wilcoxon test).* P<0.05 for the CTCT-*PTGDR* transfected cells versus CCCT- and TCCT-*PTGDR* transfected cells (ANOVA test).

Transfection assays with the above-mentioned construction vectors were performed. Culture cells were treated with 1μΜ ATRA or DMSO and collected after 24 and 48h. All haplotypic variants exhibited higher *PTGDR* expression after ATRA-treatment at 24h compared with control cells (P<0.001) (Fig 2).

We analyzed the differences on the expression of *PTGDR* among genotypic variants. Considering control cells = 1, we found: CTCT: 6.34±0.90; CCCC: 5.42±1.22; CCCT: 3.07±1.36; TCCT: 3.10±0.85 (P<0.05 for the CTCT vs. CCCT and TCCT) (Fig 2).

### ATRA stimulation promoted binding of RARα and RARβ to the *PTGDR* promoter

To interrogate whether RARs directly bound to RARE elements in ATRA-stimulated KU812 cells, a ChIP assay was carried out. The expression of RARs (α, β, γ) and RXRs (α, β, γ) on KU812 cells was analyzed, detecting only expression of RARα and RARβ mRNA (S3 Fig). Accordingly, ChIP assays were performed with RAR-antibodies.

**Fig 3:**
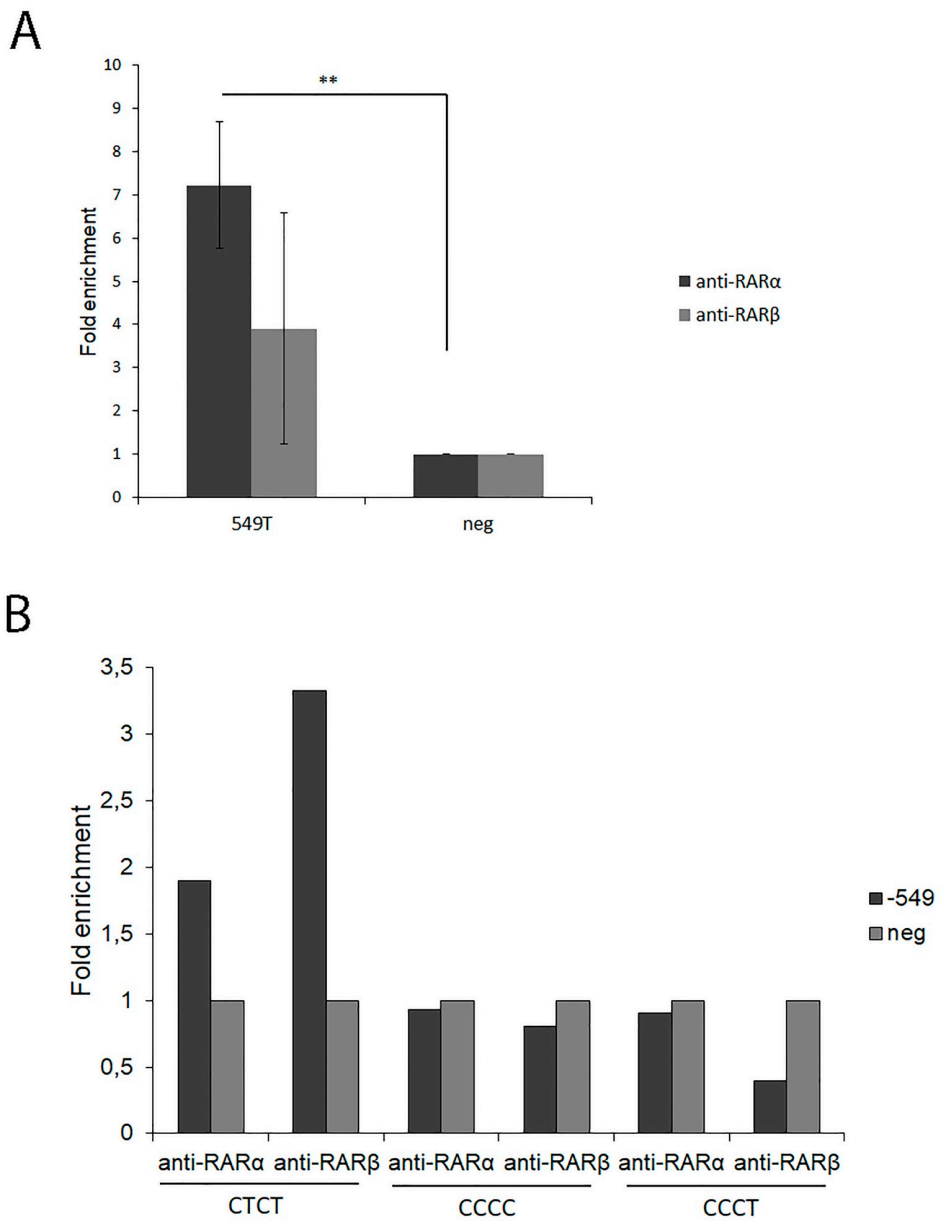
ChIP analysis of RAR isoforms on the *PTGDR* promoter. **A. KU812** cells were exposed to 1μM ATRA or DMSO for 24h. ChIP assays were performed with antibodies against RARα and RARβ. Normal Rabbit IgG was used as the negative control for immunoprecipitation. Immunoprecipitated DNA was amplified by qPCR using primers spanning the polymorphic variant -549T>C in the proximal region of the *PTGDR* promoter or using the distal *PTGDR* intron region (negative primers). Results are presented as the fold enrichment of chromatin DNA precipitated by the specific antibody compared with normal rabbit IgG. ATRA data were normalized versus DMSO. Values are means from three independent experiments. **P<0.01 for anti-RARα immunoprecipitated chromatin in the -549T promoter region versus the distal region of *PTGDR* (negative control), n = 3. **B. PBMCs** isolated from subject bearing the CTCT, CCCC or TCCC-*PTGDR* haplotype variants were subject to ChIP assays with anti-RARα and RARβ antibodies as previously described, n = 1.

ATRA stimulation promoted the binding of RARα to the proximal region of the *PTGDR* promoter (Fig 3A). No binding of RAR was detected when the distal *PTGDR* intron region was amplified (negative control). After normalizing the immunoprecipitated DNA with negative control primers, we detected fold enrichments using antibodies against RARα (7.23±1.46) and RARβ (3.90±2.69) (P<0.01 for RARα) (Fig 3A). The occupancy of a specific *PTGDR* region suggests that this element binds to both RARα and RARβ in the KU812 cell line.

### Genetic variants determined the occupancy of *PTGDR* RAREs by RARs α and β

To further determine if promoter haplotype variants influence the binding of RARα and RARβ to a RARE motif, a ChIP assay using PBMCs from individuals bearing the CTCT, CCCC and the CCCT haplotype variants was performed (Fig 3B). An increase in immunoprecipitated DNA in the CTCT compared with the CCCC and CCCT haplotype was detected when the -549T was present.

### RA up regulated the gene expression of *PTGDR* through RAR in the KU812 cell line

**Fig 4:**
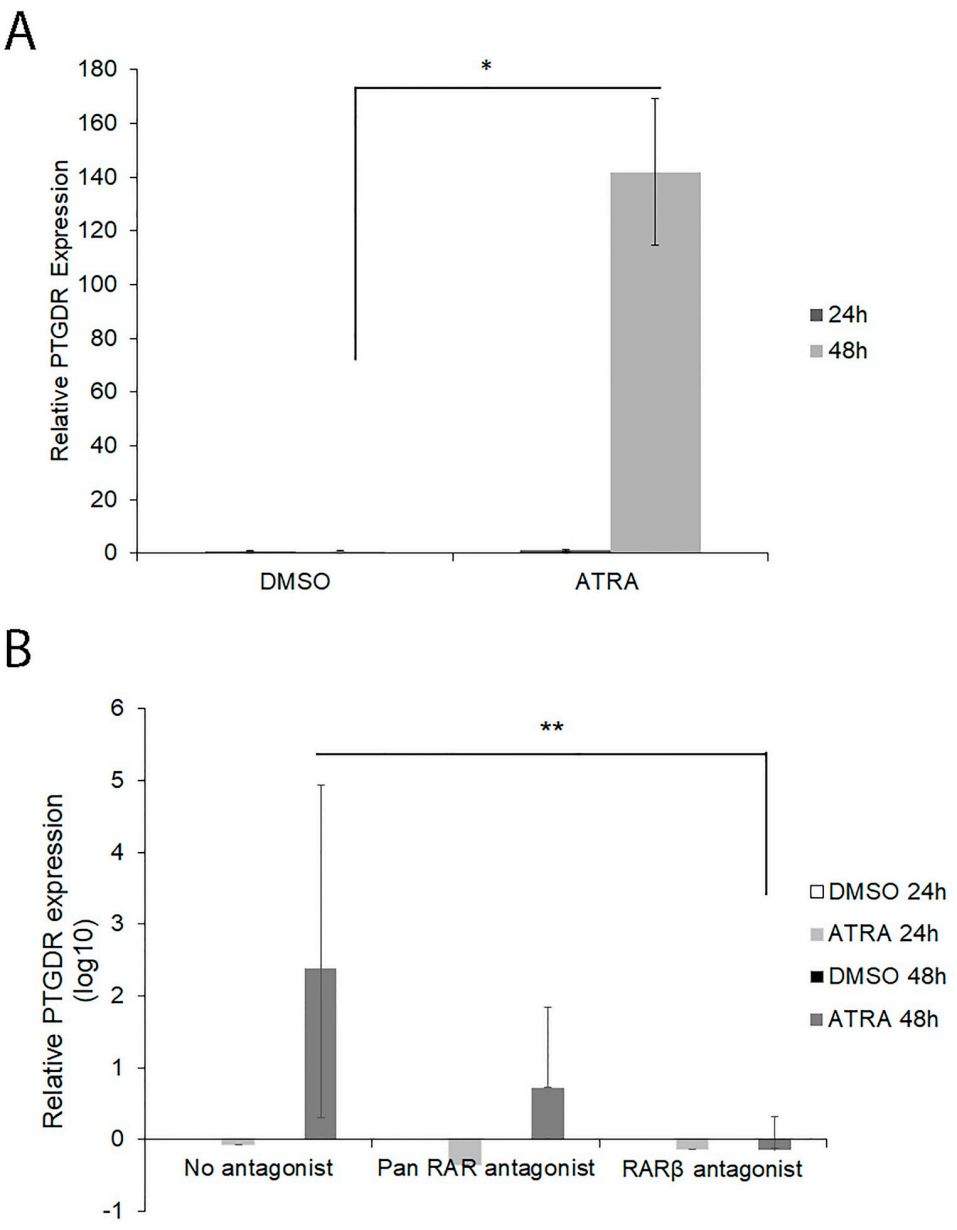
Quantitative real-time PCR analysis of *PTGDR* gene expression after ATRA or DMSO treatment in KU812 cells. **A**. KU812 cells were treated with 1 μM ATRA or DMSO and collected after 24 and 48h followed by qPCR analysis as above mentioned. Data are shown as fold increase compared with DMSO (calibrator). * P<0.05 for ATRA treated cells versus DMSO after 48h (Student’s T-test). **B**. KU812 cells were pre-treated with 1 μM of Pan RAR-antagonist (AGN 193109) or RARβ antagonist (CD 2665) 1 hour before induction with ATRA or DMSO for 24 and 48 h. Cells treated with ATRA or DMSO were used as control. Data are shown as Log10 fold increase compared with DMSO treated cells at the same conditions (calibrator). ** P<0.01 for ATRA 48h no antagonist compared with ATRA 48h RARβ antagonist (Student´s T-test).

In the KU812 cell line, the mRNA expression of *PTGDR* was upregulated by ATRA in a time-dependent manner. In the absence of RA, we could not have observed *PTGDR* expression in this cell line. However, it was strongly induced 48h after ATRA stimulation. We could barely detect expression after 24h (Fig 4A). The obtained data were: DMSO-24h: 1±0; ATRA-24h: 1.04±0.33; DMSO-48h: 1.0±0.0; ATRA-48h: 141.92±27.15 (P<0.05 for ATRA 48h compared with DMSO 48h).

Pre-treatment with 1μM of RAR antagonist (AGN 193109 or CD 2665, pan-RAR and RARβ antagonists, respectively), affected the upregulating effects of ATRA (Fig 4B). These results corroborated that the *PTGDR* promoter is likely to be a direct target of ATRA. Data in a *log10* scale were: No antagonist 48h: *log*(238.00±118.18); Pan RAR antagonist: *log*(5.26±8.21); RARβ antagonist: *log*(0.71±0.37) (P<0.01).

### The expression of *PTGDR* modified the cytokine profile in A549 cultured supernatants

A preliminary assay evaluating 27 cytokines (IL1β, IL1RA, IL2, IL4, IL5, IL6, IL7, IL8, IL9, IL10, IL12, IL13, IL15, IL17A, Eotaxin, B-FGF, G-CSF, GM-CSF, IFN-γ, IP-10, MCP-1 (MCAF), MIP-1α, MIP-1β, PDGF-BB, RANTES, TNF-α and VEGF) was performed 24 and 48h after ATRA-treatment in *PTGDR*-transfected cells.

A significant increase in IL4, IL6, IL7, IL8 and TNFα levels was detected in CTCT variant transfected cells compared with control cells. Strikingly only IL4 and IL6 increased levels were significant in both CTCT and CCCC transfected cells (Table 1). The detected cytokine levels in the supernatant were proportionate to the cytokine mRNA expression in the cells. An increase in *IL4*, *IL6* and *IL8* transcripts 48h after *PTGDR*-transfection was detected (S1 Fig). These data correlate with the cytokine measures (P<0.05). On the contrary, MIP1α and MIP1β showed significantly lower levels compared with the control condition in the CCCC variant (P<0.01).

**Table 1:** Cytokine concentration after PTGDR variants transfection in A549 cells.

Cytokine	Control	CTCT	P-value*	CCCC	P-value*
**IL4**	1	1.209±0.062	0.010	1.249±.174	0.019
**IL6**	1	1.442±0.089	<0.001	1.256±0.155	0.035
**IL7**	1	1.409±0.226	0.007	1.604±0.490	NS
**IL8**	1	1.423±0.262	0.018	1.284±0.087	NS
**IL15**	1	1.234±0.247	NS	1.247±0.178	NS
**IFNG**	1	1.215±0.069	NS	1.055±0.080	NS
**MIP1A**	1	1.152±0.167	NS	0.764±0.084	0.007
**MIP1B**	1	1.048±0.001	NS	0.840±0.058	0.012
**TNFA**	1	1.411±0.214	0.008	1.178±0.137	NS

* P<0.05 compared to control cells; NS: not significant

### ATRA-treatment modified the release of cytokines in A549 cells

After ATRA-treatment, a decrease in the concentration of IL4, IL6, Eotaxin, FGFβ, IP10, MIP1α, MIP1β, RANTES and TNFα was detected in control cells compared to DMSO. On the other hand, IL8, IL10, IL12, IL13, IFNγ, and VEGF were increased after ATRA-induction in control cells (Table 2).

**Table 2:** Cytokine concentrations after RA treatment in A549 transfected cells.

Cytokines	Control	P-value	CTCT	P-value	CCCC	P-value
**IL4**	0.6211	(0.043) †	1.4700	(0.013)*	1.5892	(0.004)**
				(0.024)†		(0.025)†
**IL6**	0.4054	(0.000) †	0.6381	(0.000)†	0.7167	(0.001)†
**IL8**	1.3269	(0.011) †	0.7894	(0.002)**	0.8625	(0.005)**
				(0.045) †		(0.013)†
**IL10**	2.2725	(0.000) †	2.1056	(0.008) †	2.1190	(0.000)†
**IL12**	2.0712	(0.001) †	1.8612	(0.000)†	2.0675	(0.000)†
**IL13**	2.1157	(0.002) †	2.7920	(0.053) †	2.4537	(0.003)†
**EOTAXIN**	0.5822	(0.031) †	1.4390	(0.001) ***	1.3361	(0.004)**
				(0.010)†		(0.092)†
**FGFB**	0.3892	(0.000)†	0.8044	(0.032)†	0.7591	(0.003)†
**IFNG**	1.6817	(0.032)†	1.3088	(0.014)†	1.2391	(0.023)†
**IP10**	0.2634	(0.000)†	0.4160	(0.000)†	0.4237	(0.000)†
**MIP1A**	0.3515	(0.000)†	0.9613	(0.010)**	0.8847	(0.015)*
**MIP1B**	0.4710	(0.002)†	0.9784	(0.016)*	0.9756	(0.017)*
**RANTES**	0.3881	(0.001)†	0.6559	(0.023)†	0.7687	(0.017)†
**TNFA**	0.6366	(0.053)†	1.5259	(0.017)*	1.9295	(0.030)*
				(0.035)†		(0.032)†
**VEGF**	2.9003	(0.000)†	2.4677	(0.000)†	2.5666	(0.000)†

***: P<0.001;

**:P<0.01;

*: P<0.05, pairwise comparisons by analysis of variance (ANOVA) compared with control cells.

^†^: P <0.05 compared to DMSO = 1, t- student test

### The effect of ATRA treatment on cytokine release is mediated by *PTGDR*

We detected differences in some of the secreted cytokines in *PTGDR* transfected cells compared with control cells. An increase in most of the above-mentioned cytokines was detected. A significant increment in IL4, Eotaxin, MIP1α, MIP1β, and TNFα was observed in contrast to a decrease in IL8 (Table 2). No significant differences were observed between the CTCT- and CCCC-*PTGDR-*variants.

These results were confirmed by qPCR, mRNA expression in A549 transfected cells after RA-treatment showed that *IL4* mRNA levels were elevated too (S2 Fig). This data correlated with cytokine measurements (P<0.01).

### *PTGDR* is stimulated by ATRA in PBMCs carrying the -549T variant

**Fig 5:**
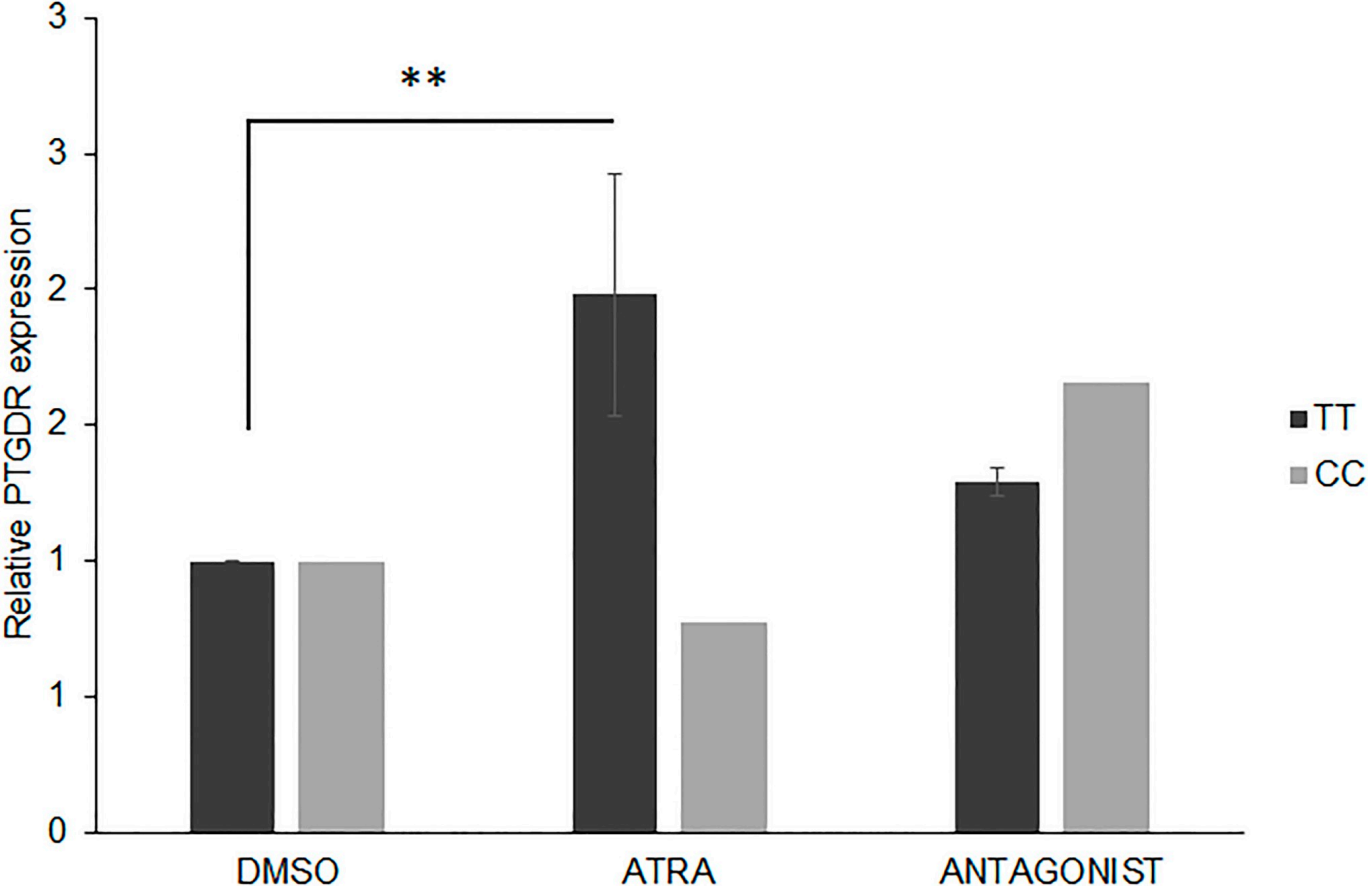
Quantitative real-time PCR (qPCR) analysis of *PTGDR* gene expression after ATRA, DMSO or antagonist treatment in PBMCs from allergic patients. PBMC carrying -549T or -549C were pre-treated with 1μM of Pan RAR-antagonist (AGN 193109) for 1h before induction with ATRA or DMSO followed by qPCR analysis of *PTGDR* as above mentioned. Cells were collected at 48h. Cells treated with DMSO were used as control. * P<0.05 for -549T PBMC after ATRA compared with DMSO, Kruskal-Wallis test. n = 3.

We performed an in vitro ATRA stimulation in PBMC cultures of two allergic patients who carried the -549T variant in the *PTGDR* promoter, and one allergic patient who carried the -549C. PBMCs were pre-treated with RARα antagonist 1h before the cells were stimulated with 1μM ATRA or DMSO. Cells were collected 48h after and RNA was isolated. The relative expression of *PTGDR* was increased in the two patients carrying the -549T variant, and this increment reverted by the RARα antagonist (*P<0.05, for ATRA compared with DMSO, Kruskal Wallis test). Conversely, the -549C patient did not show any increment of *PTGDR* after ATRA treatment. (Fig 5).

### In PBMCs carrying the -549T variant the production of Th2 cytokines was stimulated by ATRA

We analyzed the *in vitro* ATRA induced cytokine profile in PBMC cultures of two allergic patients who carried the -549T and -549C variants, respectively, as described above. mRNA of *IL4*, *IL6*, *IL8* and *IL13* were measured. The patient -549T showed increased levels of IL6, IL8 and IL13 after treatment with ATRA. This increase was attenuated when pre-treated with the antagonist, thus showing a specific effect of ATRA. On the contrary, IL4 levels were lower than DMSO. However, in the patient carrying the -549C variant ATRA treatment reduced IL4 and IL6 levels and increased IL8 levels; such changes were attenuated with pre-treatment with the antagonist (S4 Fig).

## Discussion

Epidemiological studies have related vitamin A with allergy by affecting the Th1-Th2 balance [9,14]. However, the role of RA in asthma and allergy is still controversial, [11,12,14]. We have previously reported that RA activated the *PTGDR* promoter, identifying RAREs in the promoter region and showing that transcription motifs were affected by genetic variants [17]. In the present study, we report for the first time the molecular mechanism of regulation of *PTGDR* expression by RA.

A significant increase in the *PTGDR* expression was detected in transfected cells with different haplotypic variants. CCCC showed the highest *PTGDR* expression, both at 24 and 48h. In previous reports, this haplotypic variant was associated with higher promoter activity and it was also associated to asthma [8,17,18]. Interestingly, the wt-CTCT-variant, which has been associated with lower expression, was more frequent in non-allergic controls than in allergic patients [4,6]. These data are consistent with our previous studies in which the higher expression of *PTGDR* corresponded to CCCC- and CCCT-haplotypes [18] that were mainly detected in allergic patients [21].

Noteworthy, we observed an increase in the *PTGDR* expression of all variants after ATRA-treatment, although the greatest increase was shown with the CTCT-variant. In previous studies we have reported that the promoter proximal region showed the greatest increase in luciferase activity in response to ATRA [17]. Sequence analyses revealed a RARE motif in the CTCT-variant, which exhibited the highest level of *PTGDR* expression. In this sense, we have reported a potential extra RARE motif located in the -549T>C promoter position of *PTGDR* in the CTCT-variant [17]. This extra RARE could explain the highest sensitivity of CTCT to ATRA.

We have also observed that *PTGDR* mRNA is controlled by genetic variants located within a binding site for RXR/RAR receptors. Differential allelic occupancy at -549 position of RARE determines modifications in binding, which could explain the differential regulation. Therefore, we used ChIP experiments to examine the enrichment of RARs in the *PTGDR* promoter region. ATRA stimulation significantly promoted the binding of RARα to the proximal region of *PTGDR*. Using anti-RARα we detected the biggest enrichment of immunoprecipitated DNA-protein in the sequence carrying the -549T (wt) in the KU812 cell line. In addition, we detected an increase in immunoprecipitated DNA in the PBMCs bearing the CTCT haplotype compared to those bearing the CCCC and TCCC haplotypes. The -549T>C SNP is located within the binding site for RAR transcription factors. RA increases *PTGDR* transcription and different SNPs can modify its binding through RARα and RARβ isoforms. We hypothesize that these SNPs could mediate the RA response and associate to allergic diseases.

We have also showed an upregulation of *PTGDR* after ATRA-treatment in KU812 cells. In order to explain our hypothesis of the activation of RARs by ATRA, the cells were treated with RARα and RARβ antagonists, which attenuated the response, thus confirming that the regulation of the expression of *PTGDR* by ATRA occurred through RARs. These results were confirmed in PBMCs from allergic patients who carried the -549T variant, in which the *PTGDR* expression levels were elevated after treatment with ATRA and attenuated in the presence of the antagonist. Interestingly, these responses were not observed in the patient carrying the -549C variant.

A better understanding of the influence of PTGDR and ATRA on cytokine production can help to unravel molecular bases of allergic diseases, providing the field for better clinical interventions. In this sense, we have evaluated the effect of *PTGDR-*variants on the production of cytokines in A549 cells. Thus, significant increase in the Th2 cytokine IL4, and the proinflammatory cytokines IL6, IL8 and TNFα, was detected in pCTCT-*PTGDR* transfected cells compared to un-transfected cells. However, in pCCCC-*PTGDR* transfected cells, only significant increases in IL4 and IL6 were detected. Robinson et al., observed an increase in IL4 mRNA in the bronchi of patients with atopic asthma [22]. By transcriptomic massive sequencing, we have previously reported an increased expression in *IL4RA* mRNA in B-cells of patients with allergic asthma [23]. IL4R up-regulation facilitates IL4 signaling, associated to IgE class-switch recombination [24]. It has also been reported that PGD2 increases the Th2 polarization of naïve Th cells, as evidenced by an increase of IL4 and a decrease of IFN-γ [25]. Our data corroborate the activation of IL4 in presence of *PTGDR*, which could have implications in allergic diseases.

IL6 has been involved in the synthesis of PGE2 [26], it is increased in asthmatic patients, and has been detected in the bronchoalveolar lavage fluid (BALF) of severe asthmatic patients [27]. IL6 and IL8 are pro-inflammatory mediators that have been detected in culture supernatant of A549 cells after epithelial-mesenchymal transition induction in A549 [28]. Hirano et al reported that IL8 production induced by TNF-alpha was regulated by the prostanoid DP receptor [29]. In addition, IL4, IL8 and TNFα have been related with the pulmonary function in obstructive airway diseases and could be potential markers of asthma [30].

We have previously reported that RA stimulates the promoter activity driven by the 5’-flanking region of *PTGDR* [17], which suggests that ATRA regulation occurs at a transcriptional level. In the present work, all variants exhibited higher *PTGDR* expression after ATRA treatment comparing with DMSO, corroborating previous results [17]. To interrogate the effect of RA regulation on *PTGDR* in inflammation, we analyzed cytokine levels in the supernatant of transfected cell after ATRA treatment. In control cells, we observed a decrease in the concentration of IL4 and IL6 and an increase on IL8 after treatment with ATRA. In *PTGDR* transfected cells ATRA also induced a significant decrease in IL6 and IL8, but there was a significant increase in IL4. These differences in the cytokine production between controls and transfected cells were statistically significant and seemed to be dependent on the expression of *PTGDR*. Nevertheless, we have not observed significant differences between CTCT and CCCC variants regarding the cytokine profile production after ATRA treatment. RA has been described as a key regulator of TGFβ-dependent immune responses and inhibited IL6 driven induction of Treg cell differentiation [31]. In addition, several studies have reported an anti-inflammatory activity of RA [32,33]. Furthermore, Sheffel et al, reported that ATRA significantly inhibited IL6 secretion in human B-cells stimulated with anti-CD40 plus IL4 and that the inhibition of IgE by ATRA depended on the inhibition of IL6 [34]. Babina et al. reported that ATRA increased TNFα and IL8 in mast cells while there was no impact on IL6 [35]. These data support our results in IL6 and IL8 levels. However, it is noteworthy that IL8 levels decreased in *PTGDR*-transfected cells after ATRA treatment. We also observed an increase in TNFα in transfected cells after ATRA treatment. Thus, the activation of TNFα seemed to be regulated by *PTGDR*, which is in line with reports from Hirano et al, who showed that the PGD2 receptor up-regulated the cytokine production by TNFα in THP-1 cells. Dawson et al. reported that RARα mediates human T-cell activation and Th2 production [36].

There are some limitations in our study. The use of different cell types, as lung epithelial cells, basophils, and PBMCs, can difficult the interpretation of the results, given the complexity of regulatory interactions and the possible differences among the different cellular models. Nevertheless, all cellular models point to the influence of ATRA in the expression of *PTGDR*, which is the main finding of this study, although this is not so clear in the case of cytokine production. Therefore, we believe that this particular aspect needs to be prospectively confirmed in larger series of well characterized patients.

In conclusion, our results suggest that ATRA regulates the expression of *PTGDR*, which could be important in the regulation of Th1 and Th2 responses in allergic diseases. In addition, the -549T> C polymorphism modulates the binding of ATRA to regulatory elements of the *PTGDR* promoter and this may contribute to the phenotypic differences observed in allergic patients. The potent induction of *PTGDR* by ATRA and its inhibition by specific antagonists support that these effects are mediated through RAR receptors, pointing to RAR as a potential therapeutic target in allergic diseases.

## Supporting information

S1 Appendix Methods. (DOCX)

S1 Fig qPCR analysis of cytokines gene expression. A549 cells were transfected with pCTCT-*PTGDR* and pCCCC-*PTGDR* expression vectors and with carrier DNA (control condition). Cells were collected after 24 and 48h followed by qPCR analysis of *IL4*, *IL6* and *IL8* cytokines genes. Gene expression was normalized to *GAPDH* mRNA levels. Data are shown as fold increase relative to mRNA levels for control cells. (***P<0.001 for IL6 in CTCT versus control at 24h and for IL6 in CCCC versus control at 48h; **P<0.01 for IL6 in CCCC versus control at 24h, and for IL4 in CCCC versus control at 24h; *P<0.05 for IL6 in CTCT versus control at 48h and IL8 in CCCC versus control at 24h).
(TIF)

S2 Fig qPCR analysis of cytokines gene expression after ATRA treatment. A549 transfected cells were treated with 1 μM ATRA or DMSO and collected at 48 h followed by qPCR analysis of *IL4*, *IL6*, and *IL8* cytokines genes. Cytokines gene expression was normalized relative to *GAPDH* mRNA levels. Data are shown as fold increase relative to mRNA levels for control cells and relative to DMSO. (***P<0.001 for IL4 in CTCT and CCCC transfected cells versus control at 48h; **P<0.01 for IL8 in CTCT and CCCC transfected cells versus control at 48h).
(TIF)

S3 Fig qPCR analysis of RAR isoforms gene expression after ATRA treatment. KU812 cells were treated with 1 μM ATRA or DMSO and collected at 24 and 48 h followed by qPCR analysis of RAR α, β and γ genes. RARs gene expression was normalized relative to *GAPDH* mRNA levels. Data are shown as fold increase relative to mRNA levels for DMSO.
(TIF)

S4 Fig qPCR analysis of cytokine genes expression after ATRA, DMSO or antagonist treatment in PBMCs from allergic patients. PBMC carrying -549T or -549C were pre-treated with 1 μM of Pan RAR-antagonist (AGN 193109) for 1 hour before induction with ATRA or DMSO followed by qPCR analysis cytokines IL4 (A), IL6 (B) and IL8 (C) as above mentioned. Cells were collected at 48 h. Cells treated with DMSO were used as control. n = 2.
(TIF)
