# Supplementary material for: PTGDR expression is upregulated through retinoic acid receptors (RAR) mechanism in allergy
Source: PLoS One. 2019 Apr 15;14(4):e0215086. doi: 10.1371/journal.pone.0215086 (PMC6464170; doi:10.1371/journal.pone.0215086)
Supplement: S1 Appendix — (DOCX) [file pone.0215086.s001.docx]

**S1 Appendix. Methods**

**RNA isolation and reverse transcription**

Total RNA was isolated using the RNeasy Plus Mini kit (Qiagen, Hilden, Germany) according to manufacturer’s instructions. DNAse treatment was performed using RNAse-free DNAse set (Qiagen, Hilden, Germany). Concentrations and RNA quality ratios were determined in a Nano Drop 1000 Spectrophotometer (Thermo Fisher Scientific, Waltham, MA, USA). The OD260nm/OD280nmand theOD260nm/OD230nmof samples ranged from 1.8 to 2.0. Reverse transcription (RT) was performed on 500ng of total RNA using Superscript III First-Strand Synthesis System for RT-PCR (Invitrogen, Carlsbad, CA, USA). The reaction took place in a thermal cycler (MultiGene OptiMax Thermal Cycler, Labnet International Inc., Edison, NJ, USA) in a volume of 20µl and with a single cycle and incubation periods of 65ºC for 5 min, 25ºC for 10 min, 50ºC for 50 min, 85ºC for 5 min and 37ºC for 20 min. All the analyzed samples were transcribed with the same reverse transcription reaction conditions.

**Expression analysis**

Relative qPCR was performed using LightCycler480 Instrument by SYBR Green I Master (Roche, Basel, Switzerland). Fold induction was calculated using the formula 2-(ΔΔCt) by the comparative Ct method [1]. Oligonucleotides were designed using the primer analysis software Primer 3.0 (http://bioinfo.ut.ee/primer3-0.4.0/) and assisted using Beacon Designer Software (www.premierbiosoft.comBeacon Designer). *GAPDH* reference gene primers were chosen from the panel Real Time ready Human Reference Gene (Roche Applied Science, Indianapolis, IN, USA). Primers were used at 400nM each and cDNA at 20ng in 15µl reactions. Conditions for PCR included for 10 min at 95ºC followed by 45 cycles of real-time PCR with 3 segments amplification, including 10s at 95ºC for denaturation, 10s at 60ºC for annealing, and 10s at 72ºC for polymerization. The dissociation protocol to determine the melting curve from 60°C to 95°C for each PCR product was added after thermocycling to verify that each primer pair produced only a single product. All samples showed only one melting peak, indicating that PCR generated only one amplicon and no primer/dimer formation. qPCR efficiencies were performed by amplification of a standardized dilution series of the template cDNA and were determined for each gene based on the slope of the standard curve. The qPCR efficiencies were calculated mediated the equation: E=(10-1/slope-1) x100, obtaining efficiencies values between 90% and102%. The experiments were replicated three times and every sample was performed in triplicate, with non-template controls and calibrator. All procedures were performed following MIQE guidelines [2].

**Chromatin Immunoprecipitation (ChIP) Assay**

ChIP was performed using EZ-Magna ChIP A (Millipore, Burlington, MA, USA) on KU812 cells and human PBMCs isolated from subjects bearing the *PTGDR* promoter haplotypes in which expression differences were observed [3]. KU812 cells were grown with RPMI-1640 media, 1% FBS at 1 million cells/ml. The next day, 10^7^ cells were treated with 1μM ATRA or DMSO and incubated for another 24h. Proteins cross-linked to DNA with formaldehyde 1% (v/v) (Sigma-Aldrich, Saint Louis, MO, USA) for 10 min, neutralized with glycine 5min, and washed twice with 1xPBS containing Complete EDTA-free Protease Inhibitor Cocktail (Roche Applied Science, Mannheim, Germany). Cells were lysed with cell lysis buffer and suspended in nuclear lysis buffer also both containing protease inhibitors. Chromatin was fragmented to 200-800bp using a digital ultrasonic disruptor (Branson Ultrasonic Sonifier S-250D, Thermo Fisher Scientific, Whaltham, MA, USA). Each sample was sonicated 4-times for 30s at 20% power, 1min on ice between sonications. 1% of chromatin was removed as ‘input’. For immunoprecipitation 20µl of fully suspended protein A magnetic beads (Millipore, Burlington, MA, USA) and 5µg of each Rabbit polyclonal antibody anti-RARα and anti-RARβ (Santa Cruz Biotechnology, Dallas, TX, USA) were incubated overnight at 4ºC with rotation. The total ‘input’ and a negative control of immunoprecipitation with Normal Rabbit IgG antibody (Millipore, Burlington, MA, USA) were used. After incubation, washing, elution and reverse crosslinking, DNA was purified with QIAquick PCR purification kit (Qiagen, Hilden, Germany). Eluted DNA samples were analyzed by qPCR with SYBR Green. Primers flanking the -549 position on *PTGDR* promoter and negative primers specific to a region of the *PTGDR* intron were designed. ChIP-qPCR data were normalized using the Fold-Enrichment Method with respect to the no-antibody control or signal over background. The ChIP signal is represented as the fold increase relative to the background. Experiments were run in triplicate.

**References**

1. Livak KJ, Schmittgen TD. Analysis of relative gene expression data using real-time quantitative PCR and the 2(-Delta Delta C(T)) Method. *Methods San Diego Calif* 2001;25:402–408.

2. Bustin SA, Benes V, Garson JA, Hellemans J, Huggett J, Kubista M et al. The MIQE guidelines: minimum information for publication of quantitative real-time PCR experiments. *Clin Chem* 2009;55:611–622.

3. García-Sánchez A, Marcos-Vadillo E, Sanz C, Hernández-Hernández L, Cerutti-Müller G, Marqués-García F et al. Retinoic Acid Modulates PTGDR Promoter Activity. J Investig Allergol Clin Immunol 2016;26:249–255.
