# Supplementary figures and images for: PTGDR expression is upregulated through retinoic acid receptors (RAR) mechanism in allergy
Source: PLoS One. 2019 Apr 15;14(4):e0215086. doi: 10.1371/journal.pone.0215086 (PMC6464170; doi:10.1371/journal.pone.0215086)

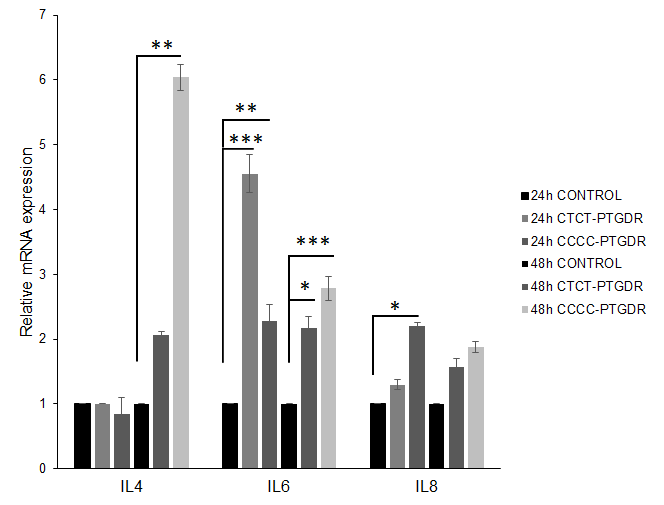

Supplement: S1 Fig — A549 cells were transfected with pCTCT-PTGDR and pCCCC-PTGDR expression vectors and with carrier DNA (control condition). Cells were collected after 24 and 48h followed by qPCR analysis of IL4, IL6 and IL8 cytokines genes. Gene expression was normalized to GAPDH mRNA levels. Data are shown as fold increase relative to mRNA levels for control cells. (***P<0.001 for IL6 in CTCT versus control at 24h and for IL6 in CCCC versus control at 48h; **P<0.01 for IL6 in CCCC versus control at 24h, and for IL4 in CCCC versus control at 24h; *P<0.05 for IL6 in CTCT versus control at 48h and IL8 in CCCC versus control at 24h). (TIF) [file pone.0215086.s002.tif]

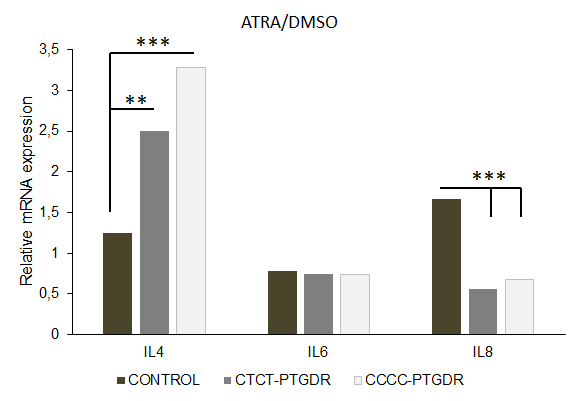

Supplement: S2 Fig — A549 transfected cells were treated with 1 μM ATRA or DMSO and collected at 48 h followed by qPCR analysis of IL4, IL6, and IL8 cytokines genes. Cytokines gene expression was normalized relative to GAPDH mRNA levels. Data are shown as fold increase relative to mRNA levels for control cells and relative to DMSO. (***P<0.001 for IL4 in CTCT and CCCC transfected cells versus control at 48h; **P<0.01 for IL8 in CTCT and CCCC transfected cells versus control at 48h). (TIF) [file pone.0215086.s003.tif]

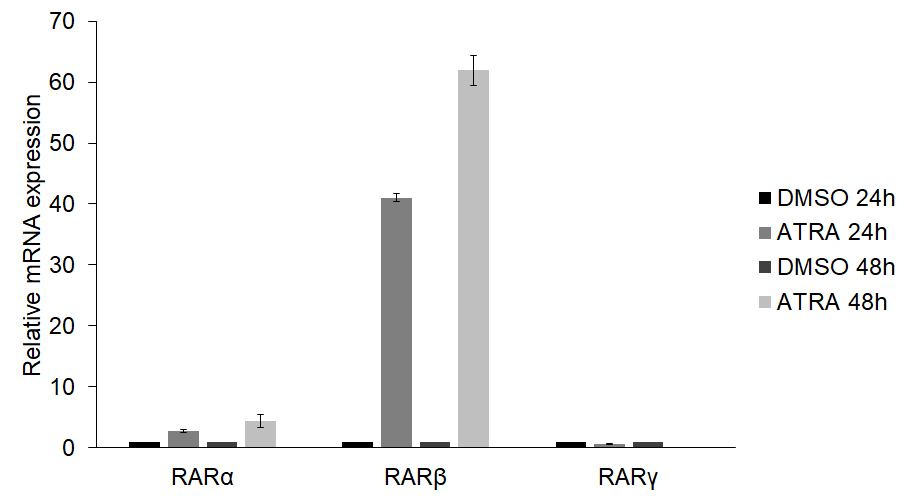

Supplement: S3 Fig — KU812 cells were treated with 1 μM ATRA or DMSO and collected at 24 and 48 h followed by qPCR analysis of RAR α, β and γ genes. RARs gene expression was normalized relative to GAPDH mRNA levels. Data are shown as fold increase relative to mRNA levels for DMSO. (TIF) [file pone.0215086.s004.tif]

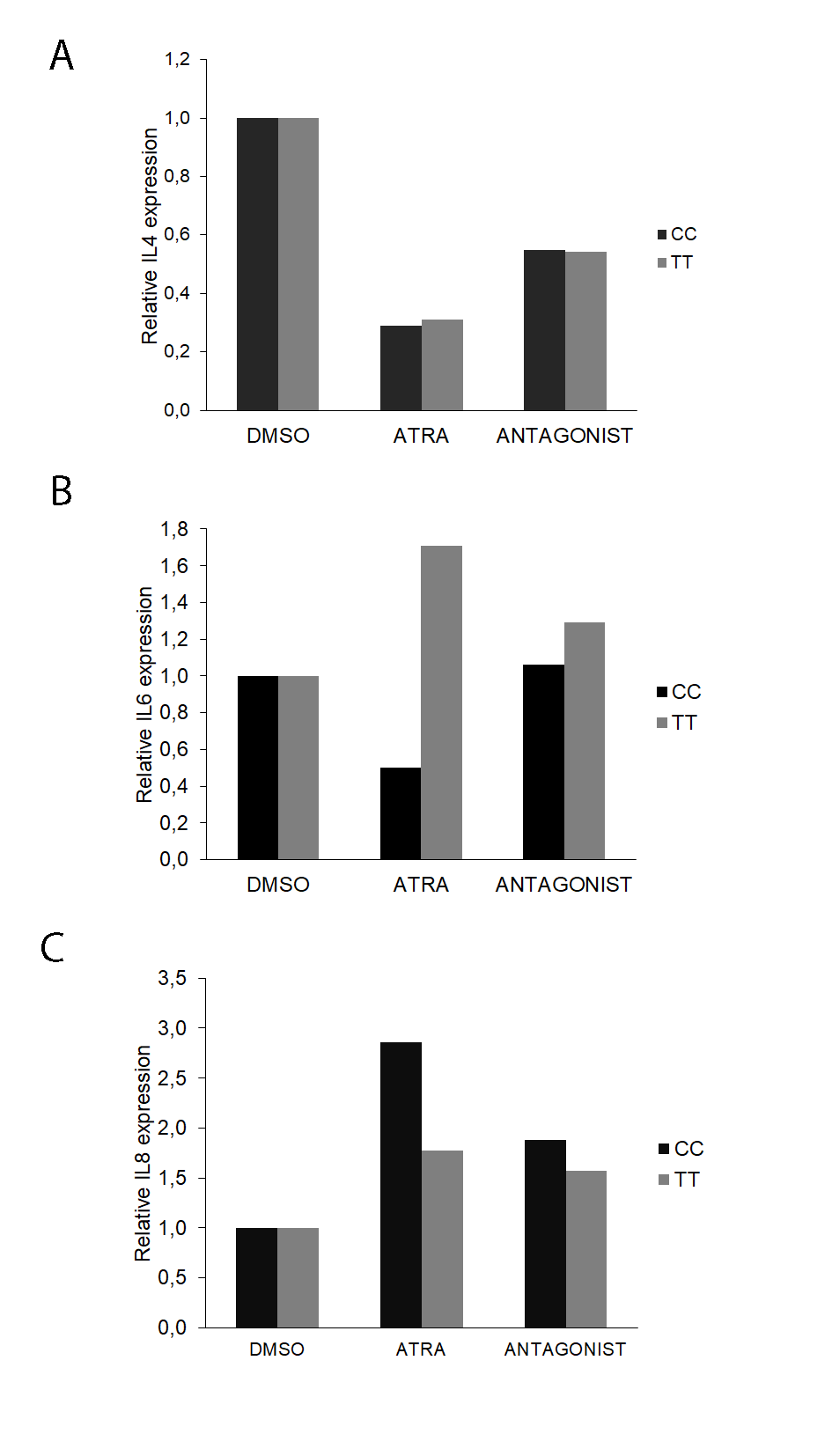

Supplement: S4 Fig — PBMC carrying -549T or -549C were pre-treated with 1 μM of Pan RAR-antagonist (AGN 193109) for 1 hour before induction with ATRA or DMSO followed by qPCR analysis cytokines IL4 (A), IL6 (B) and IL8 (C) as above mentioned. Cells were collected at 48 h. Cells treated with DMSO were used as control. n = 2. (TIF) [file pone.0215086.s005.tif]
